# Leptospirosis-induced diffuse alveolar hemorrhage: A rare case report from a non-epidemic area and literature review

**DOI:** 10.1097/MD.0000000000048131

**Published:** 2026-03-27

**Authors:** Yaofeng Yu, Songsong Hong, Zhongfa Wang, Shibo Li, Sen Zhang

**Affiliations:** a Intensive Care Unit, Putuo District People’s Hospital, Zhoushan City, Zhejiang Province, China; b Laboratory Department, Dinghai District Disease Prevention and Control Center, Zhoushan City, Zhejiang Province, China; c Infectious Diseases Department, Zhoushan Hospital, Zhoushan City, Zhejiang Province, China; d Department of Infectious Disease Prevention and Control, Dinghai District Center for Disease Control and Prevention, Zhoushan City, Zhejiang Province, China.

**Keywords:** ARDS, diffuse alveolar hemorrhage, leptospirosis, mNGS, RT-qPCR, Weil’s disease

## Abstract

**Rationale:**

Leptospirosis is an uncommon cause of severe pneumonia and diffuse alveolar hemorrhage (DAH), particularly in non-endemic areas, posing a significant diagnostic challenge. This case highlights the critical role of advanced molecular diagnostics in identifying this rare and life-threatening presentation.

**Patient concerns:**

A 65-year-old woman presented with an acute onset of high fever, chest tightness, and rapidly progressive shortness of breath.

**Diagnoses:**

The patient was initially misdiagnosed with severe community-acquired pneumonia. She subsequently developed septic shock and multiple organ dysfunction syndrome. A definitive diagnosis of leptospirosis-induced DAH was confirmed through metagenomic next-generation sequencing, reverse transcription quantitative PCR, and subsequent seroconversion shown by immunoglobulin M enzyme-linked immunosorbent assay.

**Interventions:**

Upon diagnosis, targeted antimicrobial therapy with intravenous penicillin was initiated. Supportive care included management of septic shock and lung-protective ventilation for concomitant acute respiratory distress syndrome.

**Outcomes:**

Following the confirmation of leptospirosis and initiation of targeted treatment, the patient’s condition gradually stabilized. After a course of intensive care, she made a full recovery and was successfully discharged.

**Lessons:**

This case underscores that leptospirosis can present as fulminant DAH even in non-endemic regions. A high index of suspicion, aided by epidemiological clues and the rapid application of metagenomic next-generation sequencing/reverse transcription quantitative PCR, is crucial for timely diagnosis. Prompt targeted antimicrobial therapy combined with intensive organ support is essential for a favorable outcome in severe cases.

## 1. Introduction

Leptospirosis is a zoonotic infection caused by pathogenic *Leptospira* species, typically manifesting as acute febrile illness and is transmitted through percutaneous contact with water or soil contaminated by the urine of infected animals. Clinically, leptospirosis exhibits significant heterogeneity and often follows a triphasic course: an initial bacteremic phase (days 1–7), characterized by hematogenous dissemination and hypercytokinemia; a secondary immunopathic phase (days 7–14) characterized by antibody-mediated multi-organ injury; 3) a convalescent phase, potentially complicated by post-infectious sequelae.^[1]^ While most cases of infection are subclinical or present with nonspecific viral influenza-like symptoms, severe manifestations – such as Weil’s disease – are associated with multiple organ dysfunction, with mortality rates exceeding 50%, particularly when complicated by diffuse alveolar hemorrhage (DAH) and refractory acute respiratory distress syndrome (ARDS) requiring advanced ventilatory support and intensive care support.^[2]^ This report describes a rare case of leptospirosis-associated DAH presenting as rapidly progressive respiratory failure initially misdiagnosed as severe community-acquired pneumonia. The patient subsequently developed ARDS and multiple organ dysfunction necessitating mechanical ventilation and vasopressor support. The case underscores the diagnostic complexity of atypical leptospirosis presentations and emphasizes the importance of maintaining a high index of clinical suspicion, particularly in patients with relevant epidemiological exposures. Furthermore, it highlights the critical role of integrating epidemiological assessment with advanced molecular diagnostics to facilitate timely diagnosis and targeted therapy to optimize patient outcomes.

## 2. Case presentation

Clinical data: A 65-year-old married Han Chinese female farmer from Shijia’ao Village, Donggang Street, Putuo District, Zhoushan City, Zhejiang Province, presented to the emergency department at 15:18 on September 5, 2023, with a 4-day history of febrile illness and a 1-day history of progressive chest tightness and dyspnea. The patient had no history of chronic medical conditions. Four days before admission, she developed high-grade fever (maximum temperature 39.3°C) following recent agricultural fieldwork, accompanied by intermittent dry cough, generalized fatigue, and significant lumbar myalgia, which remained untreated. On the morning of admission, the patient experienced progressive exertional dyspnea with chest discomfort, exacerbated by physical activity and accompanied by chills and limb myalgia. Initial cranial and thoracic computed tomography revealed bilateral pulmonary infiltrates suggestive of diffuse infection, along with evidence of mild chronic pulmonary inflammation, prompting admission to the infectious disease unit with a preliminary diagnosis of “pulmonary infection.”

Physical examination: On admission, the patient was alert but exhibited signs of acute respiratory distress. Vital signs were as follows: temperature 38.2°C, heart rate 106 bpm, respiratory rate 24/min, blood pressure 100/52 mm Hg, and oxygen saturation of 93% on ambient air. Physical examination showed anicteric sclerae, no lymphadenopathy or skin abnormalities, and a supple neck without thyromegaly. Pulmonary auscultation revealed bilateral coarse breath sounds, with pronounced right-sided wet rales. Cardiovascular examination showed regular rhythm without murmurs. The abdomen was soft, non-tender, and without hepatosplenomegaly or signs of peritonism. Neurological examination revealed normal motor and sensory functions and no pathological reflexes.

Laboratory results: Initial laboratory evaluations on September 5, 2023, revealed leukopenia (white blood cell count: 4.0 × 10^9^/L) with neutrophilic predominance (82.8%), thrombocytopenia (platelet count: 79 × 10^9^/L), and mild normocytic anemia (hemoglobin: 113 g/L). Inflammatory markers were significantly elevated, including C-reactive protein (CRP) at 172.37 mg/L and serum amyloid A exceeding 200 mg/L. Liver function tests indicated hyperbilirubinemia (total bilirubin: 27.5 μmol/L; direct bilirubin: 15.1 μmol/L), transaminitis (122 U/L, aspartate aminotransferase 153 U/L), and elevated alkaline phosphatase (150 U/L). Renal function was impaired, with elevated serum creatinine (119 μmol/L) and reduced estimated glomerular filtration rate (41.76 mL/min/1.73m^2^). Coagulation parameters showed prolonged prothrombin time (14.8 seconds) and elevated d-dimer (2.46 mg/L). Cardiac biomarkers revealed normal troponin I (<0.03 ng/mL) alongside mildly elevated B-type natriuretic peptide (281 pg/mL). Arterial blood gas analysis on room air demonstrated hypoxemia (pO_2_ 73.5 mm Hg) with compensatory respiratory alkalosis (pH 7.38, pCO_2_ 34.7 mm Hg), lactate level of 2.5 mmol/L, and bicarbonate of 20.1 mmol/L. SARS-CoV-2 RT-PCR testing returned negative. Blood cultures (aerobic and anaerobic, on the right) showed no bacterial growth. On September 6, 2023, nucleic acid testing for respiratory pathogens was negative for influenza A and B, respiratory syncytial virus, adenovirus, and *Mycoplasma pneumonia*; however, human rhinovirus RNA was detected. On September 7, 2023, sputum smear microscopy for acid-fast bacilli was negative. The fungal 1,3-β-d-glucan test (G experiment) was <37.50 pg/mL (reference range: 0–70), and the Aspergillus antigen (GM test) index was 0.16 (reference value < 0.50), indicating no invasive fungal infection. Repeated bilateral aerobic blood cultures remained sterile. Initial sputum cultures yielded *Candida albicans*; however, subsequent sputum cultures (on September 9, 2023), revealed only normal respiratory flora. Notably, sputum cultures on September 14 and 15, 2023, both yielded *Serratia marcescens*, indicating possible secondary nosocomial colonization or infection.

Differential diagnosis: On admission, the patient was initially diagnosed with severe pneumonia complicated by sepsis and multiple organ dysfunction syndrome; however, the etiological pathogen remained unidentified. Given the presence of high-grade fever and extensive bilateral pulmonary infiltrates, viral pneumonia was considered. Nonetheless, the negative respiratory viral nucleic acid tests and the markedly elevated procalcitonin (PCT) and CRP (infection indicators) levels, decreased the likelihood of a viral etiology. Bacterial pneumonia was subsequently regarded as the most plausible diagnosis due to the elevated inflammatory parameters. However, the radiologic pattern of diffuse, bilateral pulmonary exudative lesions was atypical for common bacterial pneumonia. Fungal pneumonia (commonly seen in immunocompromised patients and primarily characterized by bilateral interstitial lesions), was considered, particularly due to the isolated *Candida albicans* from sputum; however, the absence of immunosuppression and negative fungal biomarkers reduced its likelihood. Immune-mediated lung injury: this is considered less likely, as a marked elevation in infection biomarkers is uncommonly associated with this condition, though co-infection cannot be entirely ruled out. A limitation of this case is the lack of ancillary testing for conditions such as antineutrophil cytoplasmic antibodies-associated vasculitis, anti-glomerular basement membrane disease, systemic lupus erythematosus/antiphospholipid syndrome, or drug-induced injury, thus preventing comprehensive exclusion of these etiologies.

## 3. Treatment

Upon admission, the patient was diagnosed with severe pneumonia, and empirical anti-inflammatory treatment was initiated with Tai Neng (0.5 g every 8 hours) and intravenous methylprednisolone sodium succinate (40 mg every 12 hours). However, the patient’s oxygenation index progressively declined. Despite high-flow nasal oxygen therapy (FiO_2_ 80%, flow rate of 60 L/min), the partial pressure of oxygen (PaO_2_) was only 75.6 mm Hg, and the oxygenation index (PaO_2_/FiO_2_) dropped to 94.5 mm Hg, indicating severe hypoxemia. The patient had no underlying heart disease or high-risk factors such as hypertension or diabetes; therefore, heart failure was not considered the clinical cause of the pulmonary edema given the significant elevation in infection markers. The patient developed worsening dyspnea and hemoptysis characterized by small volumes of bright red blood-streaked sputum. Pulmonary auscultation revealed extensive bilateral crackles. Due to rapid clinical deterioration, the patient was transferred to the intensive care unit (ICU) at 17:00 on September 6, 2023 (day 2 of admission) for invasive mechanical ventilation through endotracheal intubation. At the time of ICU admission, the patient’s sequential organ failure assessment score was 13. Upon intubation, copious amounts of bright red blood-tinged sputum were observed in the oropharynx and aspirated through endotracheal suctioning, which yielded a moderate amount of blood secretions, consistent with DAH. Despite ventilatory support with high settings (FiO2% 100%, positive end-expiratory pressure (PEEP) 16 cm H_2_O, PS 15 cm H_2_O, Tidal volume 5.70 mL/kg), the patient’s peripheral oxygen saturation remained critically low (~85%). Concurrently, blood pressure decreased, necessitating vasopressor support with noradrenaline, administered through a micro-infusion pump. Given the patient’s history of agricultural work, hemoptysis, and atypical imaging findings, zoonotic infection was suspected. Metagenomic next-generation sequencing (mNGS) was immediately conducted and the local center for disease control (CDC) was notified to investigate potential zoonotic diseases. On September 6, 2023 at 24:00, the CDC provided a preliminary report indicating a suspicion of *Leptospira spp.* (spirochetes) on peripheral blood smear. The patient was immediately administered 1.6 million units of penicillin intravenously at 00:38 on September 7, 2023. One hour after the penicillin infusion, a single dose of 40 mg methylprednisolone sodium succinate was given intravenously to prevent a Jarisch–Herxheimer reaction. Following this treatment, the patient’s oxygenation improved significantly. By 04:30 on September 7, 2023, the PaO_2_/FiO_2_ ratio had reached 178 mm Hg, and hemodynamic parameters gradually stabilized, thereby avoiding the need for extracorporeal membrane oxygenation (ECMO) support. On September 7, 2023 at 11:45 the CDC confirmed the presence of *Leptospira* in the patient’s blood sample collected on September 5, 2023, establishing a definitive diagnosis of leptospirosis. On September 7, 2023, the targeted antimicrobial regimen was adjusted to include intravenous penicillin G (3.2 million units every 8 hours) and cefoperazone–sulbactam (2.0 g every 12 hours, dose-adjusted for renal function). Concurrently, a tapering course of intravenous methylprednisolone (40 mg every 12 hours for 3 days, then 40 mg daily for 2 days, and 20 mg for 1 day) was maintained for anti-inflammatory support. The total durations of therapy were 14 days for penicillin G (until September 21) and 8 days for methylprednisolone (until September 14). The sputum culture after 3 days revealed Serratia marcescens, which was sensitive to cefoperazone-sulbactam. Given the concern for a secondary pulmonary infection, the intravenous cefoperazone-sulbactam (2.0 g every 8 hours) was continued on September 9, 2023. This decision was subsequently guided by sputum cultures from September 13 and 18, which persistently grew Serratia marcescens susceptible to cefoperazone-sulbactam. The antibiotic was administered for a total duration of 17 days, concluding on September 23. On September 8, 2023, the mNGS result confirmed *Leptospira interrogans* (sequence number 44, relative abundance 57.90%), corroborating the etiological diagnosis. Serological testing was also positive for anti-leptospiral IgM and immunoglobulin G antibodies. The definitive diagnosis was established as leptospirosis complicated by DAH, severe ARDS, septic shock, multiple organ dysfunction (involving the liver and kidneys), and thrombocytopenia. Following the initiation of targeted antimicrobial and supportive therapies, the patient’s condition gradually improved. Inflammatory markers (CRP and PCT) decreased steadily, oxygenation improved, and organ functions – including renal, cardiac, and hematologic parameters (platelets counts) – gradually recovered. After 7 days of mechanical ventilation, the patient was successfully extubated and transferred to the infectious disease unit after 10 days of ICU stay. The patient continued to improve clinically and was discharged in stable condtions on September 24, 2023 (8 days after ICU transfer). The partial 16S rRNA gene sequences of *L interrogans* detected in this case have been deposited in the NCBI GenBank database under accession numbers OR852667.1 (blood) and OR852668.1 (urine). As detailed above, the patient’s condition evolved rapidly, necessitating a dynamic diagnostic and therapeutic approach. A chronological summary of these events is provided in Table 1.

**Table 1:** Key milestones in diagnosis and management.

Time point (hospital day)	Diagnostic milestone	Therapeutic interventions	Clinical basis
Pre-admission (Sep 1–4)	Onset of symptoms after fieldwork: High fever (39.3°C), dry cough, fatigue, lumbar myalgia	No treatment received	Initial symptomatic phase
Day 1 (Sep 5)	Admission: dyspnea, bilateral pulmonary infiltrates, thrombocytopenia. Initial Dx: severe pneumonia	1. Imipenem (0.5 g/8 h) 2. Methylprednisolone (40 mg/12 h)	Anti-inflammatory for potential ARDS
Day 2 (Sep 6)	ICU transfer: respiratory failure, hemoptysis; blood sample sent to CDC and for mNGS	Mechanical ventilation	Supportive care for respiratory failure; proactive testing for atypical pathogens
Day 2 (Sep 6 24:00)	CDC preliminary report – leptospira suspected on blood smear	1. Penicillin G (1.6 million U, IV) 2. Methylprednisolone (40 mg, IV)	Targeted therapy for leptospirosis; steroid to prevent Jarisch–Herxheimer reaction
Day 3 (Sep 7)	Definitive diagnosis: 1. CDC confirms Leptospira 2. mNGS: *L interrogans* (57.9% abundance) 3. Serology (IgM/IgG) positive Final Dx: leptospirosis with DAH, ARDS, septic shock, MODS	Penicillin G continued	Confirmed pathogen-directed therapy
Day 10–11	Clinical improvement: successful extubation, transferred out of ICU	Switch to oral doxycycline	Step-down therapy to complete course
Day 19	Full recovery	Discharge	Treatment completed

ARDS = acute respiratory distress syndrome, CDC = center for disease control, DAH = diffuse alveolar hemorrhage, ICU = intensive care unit, IgM = immunoglobulin M, IgG = immunoglobulin G, mNGS = metagenomic next-generation sequencing, MODS = multiple organ dysfunction syndrome.

## 4. Discussion and conclusion

Leptospirosis is a globally distributed zoonotic infection, with the World Health Organization estimating an annual incidence of 0.1 to 10 cases per 1,00,000 population, potentially rising to over 50 cases per 1,00,000 during outbreaks or among high-risk populations.^[3]^ In China, effective public health strategies and improved living conditions have significantly reduced the disease burden,^[4]^ with a consistently low national incidence from 2010 to 2022.^[5]^ In 2023, the reported incidence of leptospirosis in Zhejiang Province was 0.1262 per 1,00,000.^[6]^ Notably, the Zhoushan region had remained case-free for 4 decades, except for a single sporadic case reported in 2022,^[7]^ highlighting the rarity of the disease in this locale. The case described in this report represents the only reported leptospirosis case in Zhoushan.^[3]^

**Figure 1.**
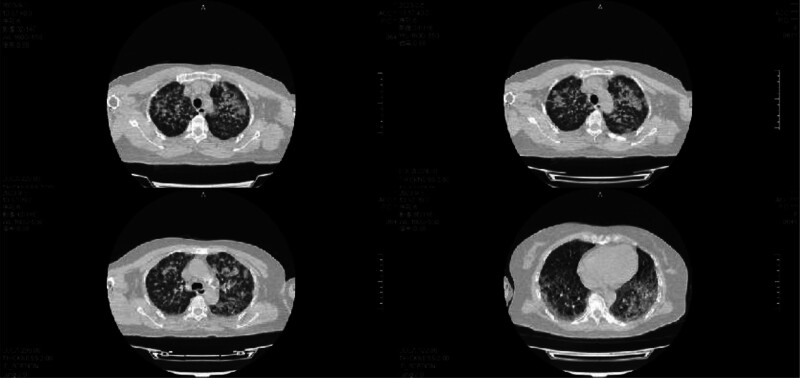
Chest CT on September 5, 2023, showed extensive bilateral patchy and flocculent high-density opacities. CT = computed tomography.

**Figure 2.**
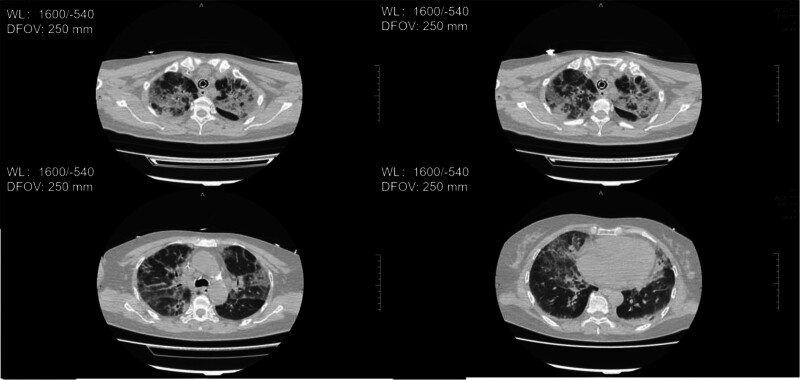
Chest CT on September 13, 2023, showed diffuse bilateral patchy and strip-like high-density opacities, indicating persistent but evolving pulmonary infiltrates. CT = computed tomography.

**Figure 3.**
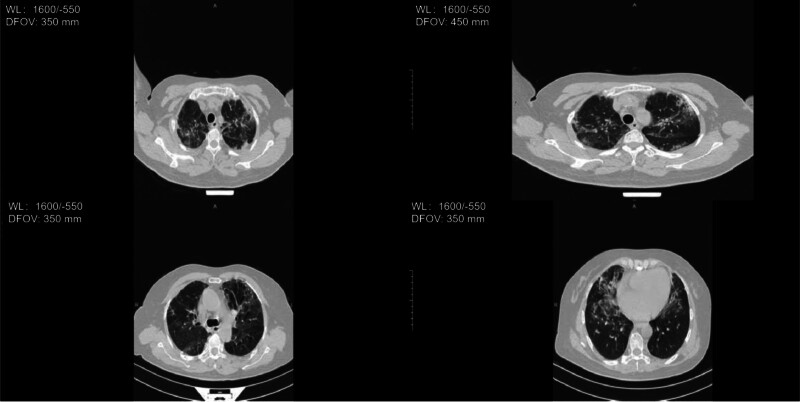
Chest CT on September 20, 2023, showed significant resolution of the bilateral patchy and strip-like high-density opacities compared with previous imaging. CT = computed tomography.

**Figure 4.**
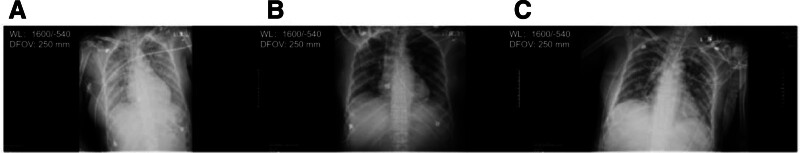
Serial bedside chest x-ray (DR), showing the progression and resolution of pulmonary infiltrates (A) On September 5, 2023, extensive bilateral patchy and high-density opacities were observed. (B) On September 9, 2023, diffuse bilateral patchy high-density opacities persisted with no significant absorption. (C) On September 12, 2023, the bilateral patchy and flocculent high-density opacities showed improvement in absorption compared with previous imaging.

**Figure 5.**
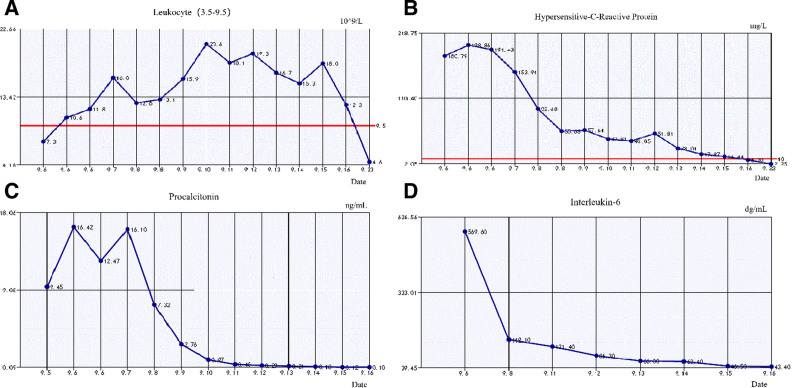
Temporal trends of infection-related biomarkers: (A) WBC count; (B) hs-CRP; (C) PCT; and (D) IL-6. WBC, hs-CRP, PCT, and IL-6 are inflammatory markers. Following the initiation of targeted antimicrobial therapy, the levels of these inflammatory markers (WBC, hs-CRP, PCT, and IL-6) showed a consistent decline. This trend correlates with the amelioration of the systemic inflammatory response and the patient’s subsequent clinical improvement. hs-CRP = high-sensitivity C-reactive protein, IL-6 = interleukin-6, PCT = procalcitonin, WBC = white blood cell.

**Figure 6.**
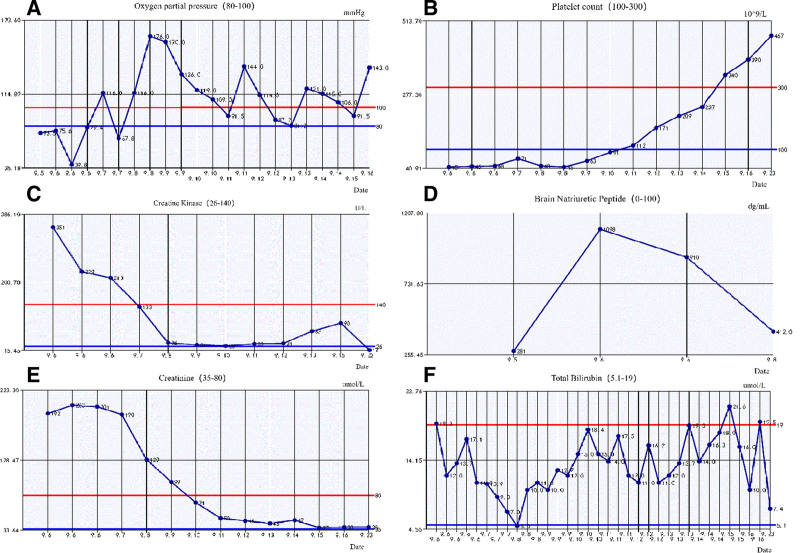
Changes in respiratory, hematologic, and organ function parameters: (A) Arterial partial pressure of oxygen (PaO_2_); (B) platelet count; (C) creatine kinase-(CK); (D) brain natriuretic peptide (BNP); (E) serum creatinine; and (F) total bilirubin. These trends visually document the patient’s fulminant clinical course and the subsequent recovery of respiratory, hematologic, cardiac, renal, and hepatic functions following the initiation of appropriate antimicrobial and supportive care. Improved PaO_2_ and oxygenation with better respiratory function. ↓Myocardial enzymes and BNP indicate improved cardiac function. ↓Cr suggests renal recovery. ↑PLT reflects better coagulation. ↓Bilirubin signals hepatic improvement. BNP = brain natriuretic peptide, CK = creatine kinase.

Clinical diagnosis of leptospirosis remains challenging owing to its protean manifestations. In this case, the patient initially presented with nonspecific symptoms – fever, fatigue, and lumbar pain – without classic clinical manifestations such as headache, conjunctival suffusion, lymphadenopathy, or calf tenderness. Upon admission, during the disease’s intermediate phase, chest computed tomography revealed extensive bilateral pulmonary infiltrates. Within 24 hours, the patient experienced rapid clinical deterioration, characterized by hemoptysis, severe hypoxemia, diffuse pulmonary rales, multiple organ dysfunction (involving cardiovascular, hepatic, and renal systems), thrombocytopenia, hypotension, and septic shock. ICU management included endotracheal intubation, mechanical ventilation with high PEEP settings, and vasopressor support. Critical epidemiological insight was gained from a retrospective exposure history, revealing recent agricultural activities in waterlogged fields converted from rice paddies to vegetable plots, adjacent to rodent-infested refuse sites. Subsequent molecular diagnostic testing (reverse transcription quantitative PCR [RT-qPCR]) targeting Leptospira 16S rRNA confirmed the diagnosis of leptospirosis, which was later speciated as *L interrogans* by mNGS. The diagnosis prompted the initiation of targeted therapy comprising cefoperazone–sulbactam, penicillin G, and adjunctive corticosteroids, which led to significant clinical improvement and stabilization of the patient’s condition. This case underscores the need for high diagnostic vigilance, thorough epidemiological assessments, and multidisciplinary collaboration when managing cases with atypical or fulminant respiratory presentations. The clinical constellation of ARDS, septic shock, and multiple organ dysfunction, was supported by imaging findings demonstrating rapidly progressive bilateral pulmonary infiltrates necessitating high-PEEP mechanical ventilation (Figs. 1–4). Laboratory findings on admission included leukopenia with significantly elevated inflammatory markers (PCT, CRP, and interleukins; Figure 5), declining PaO2, rising serum creatinine and brain natriuretic peptide levels, mild hyperbilirubinemia, and thrombocytopenia (Fig. 6).

Hemodynamic instability necessitated vasopressor support, further highlighting the severity of leptospirosis-associated DAH – a fulminant variant associated with high mortality and frequent progression to ARDS, septic shock, and multiple organ failure. While ECMO has been reported in managing severe leptospiral ARDS,^[8–11]^ this patient’s oxygenation improved with conventional supportive therapies, obviating ECMO despite an initial PaO_2_/FiO_2_ ratio below 100 mm Hg. *L interrogans*, a globally prevalent pathogenic species,^[12,13]^ invades the host through mucocutaneous breaches and disseminates hematogenously, targeting the pulmonary, hepatic, and renal microvasculature, leading to toxemic bacteremia.^[14,15]^ In this case, the diagnosis was achieved through parallel modalities: RT-qPCR detected leptospiral DNA 24 hours before mNGS. Serial mNGS testing demonstrated therapeutic response, with *L interrogans* sequence reads declining from 44 (57.90% relative abundance; Table 2) at baseline to 2 reads (5.56%) after 5 days of antimicrobial therapy, with complete clearance by day 10. Notably, sputum samples (endotracheal aspirates) remained consistently negative for leptospira, suggesting that pulmonary injury was likely secondary to immune-mediated endothelial damage rather than direct bacterial colonization. Although mNGS is a powerful tool for leptospiral detection across diverse biofluids,^[16–18]^ RT-qPCR remains the preferred diagnostic tool owing to its operational advantages including speed, high specificity, and accessibility. Serologic testing further supported the diagnosis, as it mirrored the disease course: IgM seroconversion (OD 0.995, 32.63 AU) by day 6, preceding immunoglobulin G positivity (OD 0.841, 18.72 AU) by day 8 (Table 3), consistent with mid-phase leptospiral infection at the time of clinical presentation. In this case, RT-qPCR and mNGS were pivotal for early diagnosis and timely intervention, as their rapid and highly sensitive molecular detection capabilities overcame the limitations of traditional methods. In contrast, other techniques – including serology, blood culture, and conventional PCR – were unable to meet the demands of early diagnosis due to constraints such as the serological window period, stringent culture requirements, and insufficient sensitivity.

**Table 2:** Sequencing metrics for *Leptospira interrogans* in serial blood samples.

Sequencing metric	9.6	9.11	9.15
Sequence count	44	2	0
Relative abundance (%)	57.9	5.56	0

**Table 3:** Kinetics of serum IgM and IgG antibodies during the acute phase of infection.

Parameter	9.5	9.6	9.7	9.8	9.9	9.10	9.11	9.12	9.13	9.14	9.15
IgM-OD	0.294	0.995	2.063	2.925	2.981	2.972	2.997	3.037	3.092	3.143	3.223
IgM (AU)	7.81	32.63	97.70	216.7	229.3	227.3	233.1	242.9	257.4	272.0	297.3
IgG-OD	0.149	0.224	0.433	0.841	0.867	0.845	0.921	0.988	0.95	0.976	1.035
IgG (AU)	1.18	2.31	6.11	18.72	19.95	18.90	22.78	26.92	24.47	26.12	30.34

IgM = immunoglobulin M, IgG = immunoglobulin G.

The use of adjunctive corticosteroids in severe leptospirosis, particularly in cases with predominant pulmonary hemorrhage, remains a subject of debate due to limited high-quality evidence.^[19]^ The rationale for corticosteroids in leptospirosis-associated DAH stems from the immunopathological mechanism underlying the pulmonary injury, characterized by cytokine-mediated vascular damage. While evidence remains mixed and primarily derived from observational studies and case series, some reports suggest potential benefits in rapidly progressive cases with significant pulmonary involvement. Regarding timing, corticosteroids are typically considered early in the course of severe pulmonary hemorrhage, often initiated alongside antimicrobial therapy. Dosing regimens vary, but a common approach involves methylprednisolone at 1 to 2 mg/kg/d, sometimes preceded by pulse therapy (e.g., 1 g/d for 3 days) in fulminant cases, followed by a tapered course over 1 to 2 weeks.^[20,21]^ Literature support includes a case series by Priyankara et al demonstrating improved survival with early corticosteroid administration in severe leptospirosis with pulmonary hemorrhage,^[22]^ while other studies have shown no significant benefit or potential harm.^[23]^ Given this equivocal evidence, the decision must be individualized based on clinical severity and rate of progression. In this case, the patient presented with rapid deterioration and profound hypoxemia. In this case, the patient’s critical condition – marked by rapid progression to ARDS, DAH, severe hypoxemia, and elevated interleukin-6 levels – suggested significant immunopathological injury. This prompted the empirical administration of methylprednisolone to mitigate potential cytokine-mediated lung damage. The decision was supported by several case reports and case series, which suggest that corticosteroid use in severe leptospirosis with pulmonary involvement may improve survival rates.^[20,21]^ Notwithstanding the risks associated with corticosteroid use – such as secondary infection, impaired wound healing, and hyperglycemia – the management of this life-threatening condition necessitates a risk-benefit analysis. In our assessment, the potential for stabilizing the pulmonary capillary endothelium and controlling the excessive inflammatory response compares favorably with the theoretical risks.

The antimicrobial strategy was carefully tailored to balance targeted therapy against the confirmed pathogen with the imperative to prevent or manage secondary bacterial infections, a core principle of antimicrobial stewardship. While intravenous penicillin G served as the definitive treatment for leptospirosis, the patient’s critical condition – characterized by septic shock, ARDS, and multi-organ dysfunction – significantly heightened the risk of nosocomial bacterial co-infections, a common complication in immunocompromised critically ill patients. This risk was substantiated by the subsequent sputum culture yielding *Serratia marcescens*. Therefore, the decision was made to continue the broad-spectrum coverage of cefoperazone–sulbactam concurrently. This approach embodied a pragmatic antimicrobial stewardship strategy: it provided empirical coverage against likely gram-negative pathogens while awaiting culture results, and upon identification of *S. marcescens* (which showed sensitivity to the regimen), it allowed for continued targeted treatment of the co-pathogen without escalating to a broader-spectrum agent. This managed the immediate infection risk effectively while minimizing unnecessary antibiotic exposure and the potential for fostering further resistance.

This study has limitations inherent to its design as a retrospective case report, which limits its generalizability. Furthermore, due to the critical condition of the patient, a comprehensive exclusion of alternative causes for DAH (e.g., vasculitis) was not feasible. Importantly, this case underscores the critical uncertainties in the current management of severe leptospirosis, particularly the lack of high-evidence-based standardized protocols regarding optimal antibiotic choice (e.g., penicillin G), dosing, duration, and the timing and efficacy of adjunctive corticosteroids for immunopathic phases such as DAH. Future prospective studies are warranted to establish definitive guidelines for these crucial therapeutic aspects.

In conclusion, this case underscores the diagnostic complexity and fulminant clinical course of leptospirosis-associated DAH, emphasizing the importance of early epidemiologic assessment, multidisciplinary collaboration, and timely application of molecular diagnostics for accurate pathogen identification. Early initiation of targeted antimicrobial therapy, alongside respiratory and hemodynamic support, is essential for mitigating disease progression and optimizing clinical outcomes in this rare but life-threatening condition.

## 5. Patient perspective

The experience of those days was like a nightmare. At first, I just had a fever, felt extremely weak, and had severe lower back pain. I thought it was just exhaustion from farm work and that I could tough it out. But soon, I couldn’t catch my breath-it felt like a heavy stone was pressing on my chest, and every breath became a struggle.

After being rushed to the hospital, everything happened so fast. I was in a daze, aware of being intubated and put on a ventilator, surrounded by doctors and nurses working urgently. I felt tremendous fear and helplessness, convinced I might not make it. My biggest worry was my family.

When I finally woke up in the ICU and heard the doctors say I had a rare leptospirosis infection and was improving, I began to feel a glimmer of hope. Recovery was slow – I had no strength – but I am deeply grateful that the medical team never gave up on me. They used advanced technology to identify the cause and saved my life. This brush with death has made me value my health and life even more, and I hope my case can help others with similar symptoms receive timely diagnosis and treatment.

## Acknowledgments

We thank LetPub (www.letpub.com.cn) for its linguistic assistance during the preparation of this manuscript.

## Author contributions

**Conceptualization:** Yaofeng Yu.

**Data curation:** Yaofeng Yu, Songsong Hong, Zhongfa Wang.

**Formal analysis:** Yaofeng Yu.

**Funding acquisition:** Zhongfa Wang.

**Investigation:** Sen Zhang.

**Methodology:** Shibo Li.

**Project administration:** Shibo Li.

**Resources:** Sen Zhang.

**Writing** – **original draft:** Yaofeng Yu, Songsong Hong.

**Writing** – **review & editing:** Yaofeng Yu.
